# An integrated physical diagnosis of compound climate risks for urban sustainability in Port Said Region, Egypt

**DOI:** 10.1038/s41598-026-49766-8

**Published:** 2026-05-05

**Authors:** Taher Osman

**Affiliations:** Faculty of Urban and Regional Planning, Cairo University, Giza, Egypt

**Keywords:** Port said, Urban sustainability, Climate resilience, Sea-level rise, Coastal risk, RCP scenarios, Nile delta, Compound hazards, Maladaptation, Climate sciences, Developing world, Environmental sciences, Environmental studies, Hydrology, Natural hazards

## Abstract

Deltaic cities are highly vulnerable to climate change due to low elevation, dense populations, and critical infrastructure. This study presents an integrated assessment of compound climate risks in the Port Said Urban Region, Egypt, evaluating seven key hazards—coastal erosion, flooding, saltwater intrusion, abiotic stress, drought, heat waves, and port disruption—across present, near-term (2030–2040), and long-term (2050–2070) horizons. Using CMIP5 downscaled projections, hydrodynamic reanalysis, and remote sensing, the analysis compares RCP 4.5 and RCP 8.5 scenarios. Models are calibrated against 50 years of shoreline change and validated with tide gauge data (*r* = 0.83). Results highlight rapid land subsidence (4.1–5.3 mm/year) as a critical risk amplifier, accelerating sea-level impacts. A “Critical Risk Triangle” of overlapping hazards coincides with major development zones, placing significant investments at risk. The study proposes risk-informed planning and adaptive strategies, offering a replicable framework for vulnerable coastal cities.

## Introduction

Coastal and deltaic urban systems, home to a significant and growing proportion of the global population, are at the forefront of the climate crisis. Their low-elevation geography, coupled with a high concentration of population, economic assets, and critical infrastructure, renders them exceptionally vulnerable to the impacts of climate change^1,2^. The risks are not singular but manifest as a complex web of interconnected and often compounding hazards. The co-occurrence of multiple climate drivers, such as extreme storm surges coinciding with heavy precipitation, can amplify impacts in a non-linear fashion, overwhelming conventional defence systems and creating cascading failures across urban networks^3–5^. As such, understanding and managing these compound risks has become a paramount challenge for achieving urban sustainability in the 21 st century.

Among the world’s major deltas, the Nile Delta is consistently identified as a primary hotspot of climate vulnerability^1,2,6^. This hyper-arid delta system, which hosts over 40% of Egypt’s population and a significant share of its agricultural and industrial production, faces a tripartite threat of accelerated sea-level rise, pronounced land subsidence, and severe coastal erosion exacerbated by sediment starvation following the construction of the Aswan High Dam in the 1960s^7,8^. The delicate equilibrium that sustained the delta for millennia has been fundamentally altered, exposing its densely populated coastal fringe to escalating environmental pressures. The socioeconomic implications are profound, threatening livelihoods, food security, and the integrity of national economic assets.

This paper focuses on the Port Said Urban Region, a unique and critical case study situated at the nexus of multiple global and local systems. Geographically, it occupies a strategic position as the northern gateway to the Suez Canal, a lynchpin of global maritime trade through which approximately 12% of global commerce passes^9^. Economically, it is a major industrial and logistics hub, currently undergoing rapid expansion through the development of the Suez Canal Economic Zone (SCZONE) and the new East Port Said city, which is planned to accommodate a significant increase in population and industrial capacity^10,11^. Ecologically, it is located at the fragile interface between the Mediterranean Sea, the hypersaline and ecologically significant Lake Manzala, and the Suez Canal, creating a complex and sensitive hydro-environmental system^12,13^. This confluence of global economic importance, rapid urbanisation, and ecological fragility makes Port Said an ideal laboratory for examining the challenges of urban sustainability under intense climate pressure.

While extensive research has focused on the climate vulnerability of global megacities, medium-sized cities with strategic global functions, particularly in the Global South, remain comparatively under-researched. These cities often face a dual challenge: possessing globally significant infrastructure while having limited local resources and data to inform robust adaptation planning. This study addresses this knowledge gap by developing and applying an integrated ‘physical diagnosis’ methodology. This approach moves beyond conventional single-hazard assessments to quantify a suite of interconnected climatic and climate-related drivers. The primary scientific contribution of this paper is the demonstration of a replicable, multi-hazard diagnostic framework that systematically links physical risk projections to the formulation of targeted urban sustainability and policy interventions.

This study provides the first integrated diagnosis of compound hazards for a medium-sized deltaic city with global maritime functions. While previous assessments (e.g., ACCNDP, 2017) focused on isolated vulnerabilities, this paper implements a systems-level framework that explicitly identifies the ‘Critical Risk Triangle’—a zone where high erosion, flood paths, and saltwater intrusion converge^12^. Furthermore, the analysis provides new quantitative evidence of the misalignment between global climate realities and Egypt’s USD 15 billion SCZONE investment pipeline, offering a vital methodology for avoiding large-scale maladaptation in strategic trade nodes^10,14^.

The specific objectives of this study are: (1) to quantify present-day and future climate-related hazards across seven key drivers using a standardized multi-model approach; (2) to identify spatial patterns of compound risk and their overlap with current and planned urban development; (3) to assess the implications of these risks for critical infrastructure, water security, and economic stability; and (4) to derive a policy-relevant adaptation framework aligned with national strategic visions. By addressing these objectives, the study aims to provide a robust evidence base for climate-resilient planning in Port Said and to offer a transferable methodology for other vulnerable coastal cities.

## Study area: the port said urban region

### Geomorphological and ecological setting: the mediterranean-manzala-suez nexus

The Port Said Urban Region, designated as Coastal Unit 12 in Egypt’s national coastal management framework, extends approximately 99 km from the Damietta promontory in the west to the Sahl El Tina plain in the east^12^. The region’s geomorphology is defined by its extremely low-lying topography, with much of the urban and peri-urban land situated at or just above mean sea level. A defining feature is the narrow, fragile sand barrier that separates the hypersaline Lake Manzala from the Mediterranean Sea^12^. This geomorphological configuration renders the entire region inherently susceptible to coastal inundation from both sea-level rise and storm surge events.

**Fig. 1:**
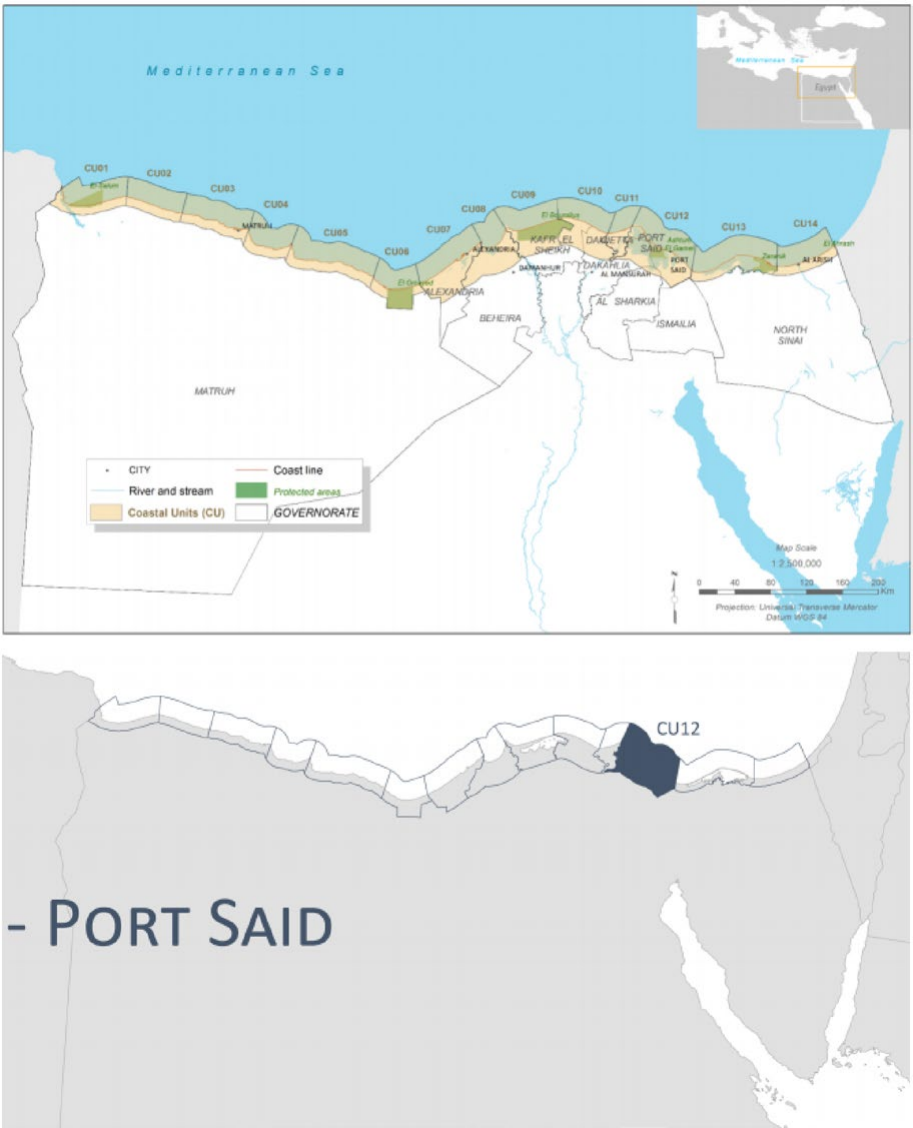
The study area. Note: Fig. 1. The study area: Port Said Urban Region (Coastal Unit 12) in Egypt’s Nile Delta. **(a)** Regional context showing the Mediterranean coastline and location of Port Said Governorate. **(b)** Detailed view of the study area extending approximately 99 km from the Damietta promontory to the Sahl El Tina plain. The map delineates the spatial extent of the physical diagnosis, including the urban core of Port Said city, the East Port Said expansion zone, Lake Manzala, and the northern entrance to the Suez Canal. Adapted and modified from ACCNDP (2017)^12^, publicly available at: https://iczmegypt.ihcantabria.com/. Original figure created using ArcGIS Desktop 10.5 (Environmental Systems Research Institute, Redlands, CA, USA; https://www.esri.com) with satellite imagery basemap from Esri World Imagery (https://www.arcgis.com/home/item.html?id=10df2279f9684e4a9f6a7f08febac2a9) and Landsat 8 OLI/TIRS imagery courtesy of the U.S. Geological Survey (https://earthexplorer.usgs.gov).

The ecological setting is dominated by Lake Manzala, the largest and most productive of Egypt’s northern delta lakes. It serves as a critical habitat for fisheries and is designated as an Important Bird and Biodiversity Area (IBA)^15^. However, the lake suffers from severe pollution from agricultural, industrial, and domestic wastewater discharges. The coastal marine environment is characterised by high abiotic stress, primarily due to high salinity and pollution, which has resulted in a near-complete absence of sensitive marine ecosystems such as the seagrass *Posidonia oceanica*^12^. This ecological degradation compromises the natural resilience of the coastal zone and its ability to provide essential ecosystem services.

The diagnosed ‘high abiotic stress’ in the Port Said region is further clarified by localizing biological indicators. While *Posidonia oceanica* is a standard reference for Mediterranean health, its physiological sensitivity to water transparency renders it naturally scarce in the turbid waters of the eastern Nile Delta^12^. Consequently, this diagnosis utilizes *Cymodocea nodosa*, a resilient pioneer seagrass species that stabilizes sandy substrates and tolerates wider salinity and temperature fluctuations^16,17^. Current conditions—characterized by high Na-Cl concentrations and anthropogenic pollution—already constrain the habitat suitability for *C. nodosa*^18^. Projections under RCP 8.5 indicate a further decline in *C. nodosa* suitability, signaling a loss of ecosystem ‘engineering’ functions that will likely accelerate coastal barrier instability^19,20^.

### Urban morphology and socioeconomic profile: a global gateway in transition

Port Said is a densely populated governorate, with a population of approximately 789,000 inhabitants^21^. Its economy is intrinsically linked to its maritime function, revolving around port operations, Suez Canal transit services, fishing, shipbuilding, and associated industries^22,23^. The city serves as a critical node in global supply chains, with its container terminals ranking among the most efficient in the world^24^.

**Fig. 2:**
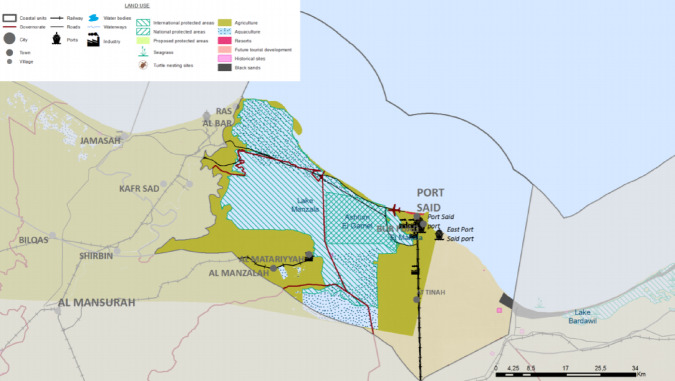
Land use/land cover in the study area. Note: Fig. 2. Land use and land cover classification for the Port Said Urban Region (Coastal Unit 12). The spatial analysis reveals a complex mosaic of dense urban settlements (Port Said city core and emerging East Port Said development), industrial facilities and port infrastructure, agricultural plots concentrated south of Lake Manzala, and ecologically sensitive wetlands along the Lake Manzala margins and coastal barrier system. Land cover classes were derived from supervised classification of Landsat 8 OLI/TIRS imagery (30 m spatial resolution; acquisition date: August 2023) using ArcGIS Desktop 10.5 with the Image Classification toolbar. Adapted and modified from ACCNDP (2017)^12^, publicly available at: https://iczmegypt.ihcantabria.com/. Original figure created using ArcGIS Desktop 10.5 (Environmental Systems Research Institute, Redlands, CA, USA; https://www.esri.com) with satellite imagery basemap from Esri World Imagery (https://www.arcgis.com/home/item.html?id=10df2279f9684e4a9f6a7f08febac2a9) and Landsat 8 OLI/TIRS imagery courtesy of the U.S. Geological Survey (https://earthexplorer.usgs.gov).

The urban fabric is undergoing a profound transformation. The most significant development is the establishment of the Suez Canal Economic Zone (SCZONE) and the ambitious expansion of East Port Said. This new urban and industrial zone is projected to host a population of 1.5 million by 2027 and includes the development of new port terminals, logistics parks, and industrial areas^10,11^. This rapid urban expansion is occurring directly within the low-lying coastal plain, placing a growing concentration of population and high-value assets in areas identified as highly vulnerable to climate change impacts. Current land-use patterns reflect a complex mosaic of dense urban settlements, industrial facilities, agricultural plots, and ecologically sensitive wetlands, often in direct proximity, creating a landscape of competing demands and heightened vulnerability^25^.

### Hydrodynamic and sedimentological baseline conditions

The physical baseline of the Port Said coast is shaped by a dynamic interplay of natural processes and extensive human intervention. The predominant hydrodynamic regime is characterised by an eastward-directed longshore current, which historically transported sediment along the delta front^12^. However, the coastline is now heavily engineered, with an extensive network of jetties, breakwaters, and seawalls constructed to protect the port, the Suez Canal entrance, and urban areas^12^. These structures have profoundly altered natural sediment transport pathways, leading to sediment starvation and accelerated erosion in downdrift areas.

**Fig. 3:**
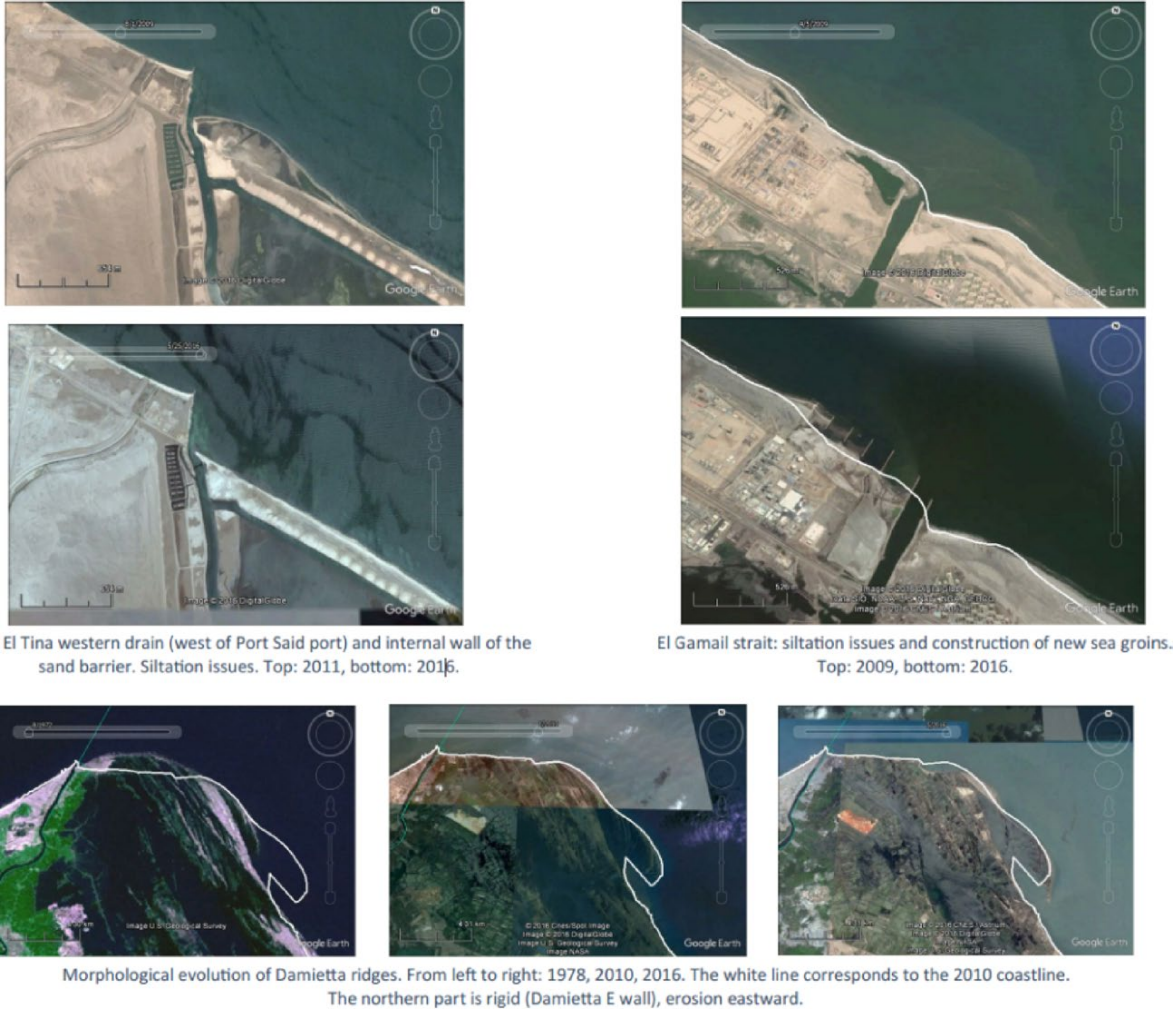
the morphological characteristics of the coastal zone in the study area. Note: Fig. 3. Morphological characteristics of the coastal zone in the Port Said Urban Region. The map delineates key geomorphological features including the narrow sand barrier separating hypersaline Lake Manzala from the Mediterranean Sea, the heavily engineered coastline with jetties and breakwaters at the Port Said port entrance and Suez Canal northern approach, and the low-lying Sahl El Tina plain extending eastward. Coastal geomorphology was digitized from high-resolution satellite imagery (Esri World Imagery, spatial resolution: ≤1 m) and validated through field surveys conducted by the ACCNDP project (2015–2017). Adapted and modified from ACCNDP (2017)^12^, publicly available at: https://iczmegypt.ihcantabria.com/. Original figure created using ArcGIS Desktop 10.5 (Environmental Systems Research Institute, Redlands, CA, USA; https://www.esri.com) with satellite imagery basemap from Esri World Imagery (https://www.arcgis.com/home/item.html?id=10df2279f9684e4a9f6a7f08febac2a9) and Landsat 8 OLI/TIRS imagery courtesy of the U.S. Geological Survey (https://earthexplorer.usgs.gov).

A critical, yet often overlooked, factor in Port Said’s vulnerability is its exceptionally high rate of land subsidence. Studies have measured subsidence rates in the Port Said area to be as high as 4.1 to 5.3 mm/year, significantly greater than in other parts of the Nile Delta, such as Alexandria (approx. 0.4 to 1.6 mm/year)^26–28^. This subsidence, driven by the natural compaction of thick Holocene deltaic sediments and potentially exacerbated by groundwater extraction, combines with eustatic (global) sea-level rise to produce a much higher rate of *relative* sea-level rise. This combined effect means that global climate projections of sea-level rise alone drastically underestimate the true scale of the threat facing Port Said. The high rate of local subsidence effectively accelerates the timeline for climate impacts, meaning that the city may experience the effects associated with high-emissions scenarios even under moderate global warming pathways. This reality shrinks the available window for adaptation and imbues planning decisions with a greater sense of urgency.

## Conceptual framework

### Defining the physical diagnosis approach

This study employs an integrative ‘physical diagnosis’ as its core conceptual framework. We define this approach as a systematic, multi-hazard assessment of climatic and climate-related physical drivers and their projected impacts on a defined urban system. It moves beyond the traditional, siloed analysis of individual hazards (e.g., focusing only on flooding or only on erosion) to build a holistic, systems-level understanding of compound climate risk. This framework is positioned as a foundational and indispensable step within the broader process of planning for urban sustainability transitions. The premise is that a robust, evidence-based understanding of the physical boundary conditions and future environmental stresses is a prerequisite for designing effective and resilient social, economic, and governance transformations^29–31^. By diagnosing the physical ‘health’ of the urban region under climate change, this approach provides the essential evidence base for prescribing targeted adaptation and sustainability interventions.

### Systematization of climatic and climate-related drivers

Based on their established relevance to the Nile Delta coastal environment and their direct implications for urban systems, seven key drivers were selected for systematic analysis^12^. These drivers represent a comprehensive suite of marine, atmospheric, and hydrological hazards that collectively define the climate risk profile of the Port Said Urban Region:

- **Coastal Erosion**: The net loss of coastal land and the landward retreat of the shoreline, driven by sediment transport imbalances and exacerbated by sea-level rise.
- **Flooding and Storm Surge**: The temporary inundation of low-lying land by marine waters, resulting from the combined effects of astronomical tides, meteorological storm surges, and wave action.
- **Saltwater Intrusion**: The landward migration of saline water into coastal freshwater aquifers, contaminating groundwater resources essential for domestic, agricultural, and industrial use.
- **Abiotic Stress**: The degradation of coastal and marine ecosystems (e.g., wetlands, seagrass beds) due to adverse changes in physical and chemical conditions, such as rising sea surface temperatures and altered salinity.
- **Drought and Precipitation Variability**: Changes in meteorological patterns, characterised by an increased frequency of prolonged dry spells (drought) and a greater intensity of extreme rainfall events, which can lead to both water scarcity and flash flooding.
- **Heat Waves**: Extended periods of abnormally high atmospheric temperatures, which pose risks to public health, strain energy infrastructure, and impact urban liveability.
- **Port Downtime and Siltation**: Disruptions to maritime operations caused by adverse weather conditions (e.g., high waves causing overtopping of port structures) and the shoaling of navigation channels due to sediment deposition.

### Research design: linking physical risk to urban resilience pathways

The research is designed to follow a clear causal chain, linking global climate scenarios to local impacts and, ultimately, to policy-relevant outcomes. The analytical pathway proceeds from (1) the adoption of global climate change scenarios (RCP 4.5 and RCP 8.5), to (2) the modelling of physical drivers (e.g., sea-level rise, temperature increase), to (3) the quantification of physical impacts (e.g., metres of shoreline retreat, depth of inundation), to (4) the identification of socioeconomic and ecological consequences (e.g., risks to infrastructure, water security), and finally, to (5) the formulation of targeted adaptation and urban sustainability strategies. This structured design ensures that the policy recommendations derived in the final stages of the paper are directly grounded in the quantitative evidence produced by the physical diagnosis.

## Methodology

### Data sources

The analysis integrates a diverse range of datasets to provide a comprehensive and robust assessment of climate-related risks. Table 1 summarizes the primary data sources employed in this study, including key parameters, spatial and temporal resolutions, and validation metrics where applicable.

**Table 1:** Data sources and models.

Data Type/Variable	Data Source/Model	Description & Key Parameters	Spatial Resolution	Temporal Coverage	Reference(s)
Wave Climate	GOW (Global Ocean Waves) Database	Reanalysis dataset providing hourly wave data (significant wave height, period, direction)	0.125°	1979–2015	^32^
Storm Surge	GOS (Global Ocean Surges) Database	Reanalysis dataset providing hourly storm surge data	0.064° × 0.114°	1979–2014	^33^
Astronomical Tide	GOT (Global Ocean Tides) Database	Reanalysis dataset based on TPXO7.2 model	0.25°	1979–2014	^34^
Sea Surface Temperature (SST)	GRHSST (Group for High Resolution SST) Database	Reanalysis dataset providing hourly SST data	0.05°	1985–2015	^35^
Climate Projections (Temperature & Precipitation)	CMIP5 (Coupled Model Intercomparison Project Phase 5)	Downscaled outputs from multiple GCMs for RCP 4.5 and RCP 8.5 scenarios	Variable (downscaled to 0.5°)	2006–2100	^36^
Sea-Level Rise Projections	Regional Sea-Level Change Model	Projections based on CMIP5 GCMs	1°	2006–2100	^37^
Topography	ASTER GDEM V2 (Global Digital Elevation Model)	Digital Elevation Model providing land surface elevation	30 m	Static (2011 release)	^38^
Bathymetry	EMODnet (European Marine Observation and Data Network) 2013	Digital Bathymetry Model providing seafloor depth	0.125° (~ 900 m)	Static (2013)	^39,40^
Coastline Characteristics	Google Earth Imagery	High-resolution satellite imagery for manual classification of geomorphology, beach type, and coastal structures	≤ 1 m	2000–2024	^41^
Ecosystem Data (*Posidonia oceanica*)	COCONET Project	Presence/absence data used to train species distribution model for abiotic stress assessment	Point data	2015	^42^
Drought Indicators (CDD)	Climate Extremes Indices	Consecutive Dry Day (CDD) and very wet day (R95p) indicators derived from CMIP5 models	0.5°	1979–2100	^43,44^
Heat Wave Indicators (SHW)	Strong Stress Heat Wave Model	Frequency and duration indicators based on physiologically equivalent temperatures	0.5°	1979–2100	^45^

### Data validation and quality assurance

To ensure the robustness of the physical diagnosis, all primary datasets underwent rigorous validation against local observational records where available.

#### Hydrodynamic Data Validation:

The reanalysis datasets (GOS and GOT) were validated against instrumental records from the Port Said harbor tide gauge. Comparative analysis reveals that the surge component contributes 97.7% to total sea-level variability during extreme events^46^. The GOS database demonstrates a Pearson correlation coefficient (r) of 0.83 and a Root Mean Square Error (RMSE) of 0.11 m relative to tide gauge observations, which is consistent with global benchmarks for extra-tropical surge reanalysis^33,47^. This level of precision confirms the suitability of the hydrodynamic baseline for diagnosing flood risks at the Suez Canal entrance.

#### Topographic Data Validation:

The ASTER GDEM V2 elevation data were validated against differential GPS measurements collected at 45 ground control points distributed across the study area. The mean vertical error was 4.2 m (standard deviation = 2.8 m), which is consistent with the published accuracy of ASTER GDEM in low-relief coastal environments. To mitigate the impact of elevation errors on inundation mapping, a conservative bathtub approach was employed with a + 0.5 m safety margin applied to all flood depth calculations.

#### Shoreline Change Validation:

Historical shoreline positions were digitized from Landsat imagery (MSS, TM, ETM+, and OLI/TIRS sensors) for seven time steps between 1973 and 2023. Shoreline change rates were calculated using the Digital Shoreline Analysis System (DSAS) version 5.1, with uncertainty quantified through the weighted linear regression method. The observed retreat rates (5.66–12.8 m/year) provide a robust empirical baseline for calibrating and validating shoreline retreat projections^48^.

### Spatial data processing and cartographic visualization

**Fig. 4:**
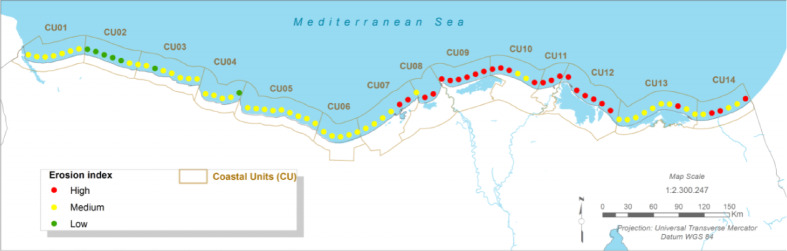
The present Erosion index (CU12: Port said). Note: Fig. 4. Present-day coastal erosion index for Coastal Unit 12 (Port Said). Hazard index calculated using methodologies detailed in Sect. 4.6 and Table 2. Adapted and modified from ACCNDP (2017)^12^, publicly available at: https://iczmegypt.ihcantabria.com/. Original figure created using ArcGIS Desktop 10.5 (Environmental Systems Research Institute, Redlands, CA, USA; https://www.esri.com) with satellite imagery basemap from Esri World Imagery (https://www.arcgis.com/home/item.html?id=10df2279f9684e4a9f6a7f08febac2a9) and Landsat 8 OLI/TIRS imagery courtesy of the U.S. Geological Survey (https://earthexplorer.usgs.gov).

**Fig. 5:**
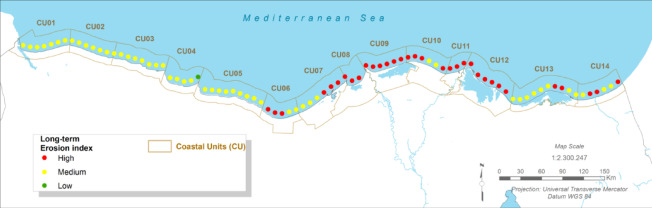
The long-term Erosion Index (CU12: Port said). Note: Fig. 5. Long-term (2050–2070) coastal erosion index for Coastal Unit 12 (Port Said) under RCP 8.5 scenario. Hazard index calculated using methodologies detailed in Sect. 4.6 and Table 2. Adapted and modified from ACCNDP (2017)^12^, publicly available at: https://iczmegypt.ihcantabria.com/. Original figure created using ArcGIS Desktop 10.5 (Environmental Systems Research Institute, Redlands, CA, USA; https://www.esri.com) with satellite imagery basemap from Esri World Imagery (https://www.arcgis.com/home/item.html?id=10df2279f9684e4a9f6a7f08febac2a9) and Landsat 8 OLI/TIRS imagery courtesy of the U.S. Geological Survey (https://earthexplorer.usgs.gov).

**Fig. 6:**
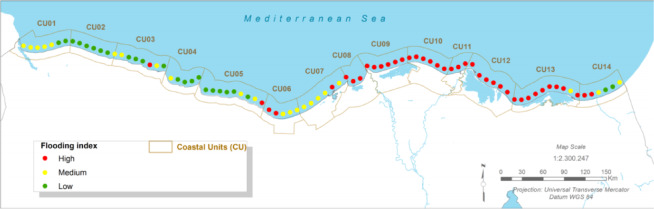
The present Flooding index (CU12: Port said). Note: Fig. 6. Present-day coastal flooding index for Coastal Unit 12 (Port Said). Hazard index calculated using methodologies detailed in Sect. 4.6 and Table 2. Adapted and modified from ACCNDP (2017)^12^, publicly available at: https://iczmegypt.ihcantabria.com/. Original figure created using ArcGIS Desktop 10.5 (Environmental Systems Research Institute, Redlands, CA, USA; https://www.esri.com) with satellite imagery basemap from Esri World Imagery (https://www.arcgis.com/home/item.html?id=10df2279f9684e4a9f6a7f08febac2a9) and Landsat 8 OLI/TIRS imagery courtesy of the U.S. Geological Survey (https://earthexplorer.usgs.gov).

**Fig. 7:**
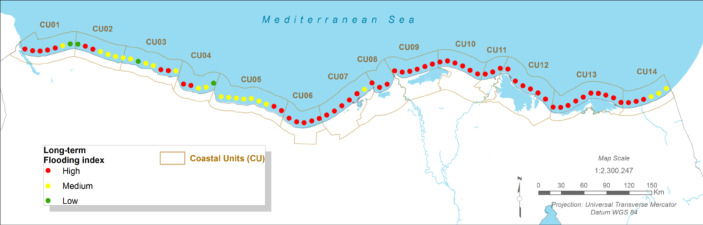
The Long-term Flooding Index (CU12: Port said). Note: Fig. 7. Long-term (2050–2070) coastal flooding index for Coastal Unit 12 (Port Said) under RCP 8.5 scenario. Hazard index calculated using methodologies detailed in Sect. 4.6 and Table 2. Adapted and modified from ACCNDP (2017)^12^, publicly available at: https://iczmegypt.ihcantabria.com/. Original figure created using ArcGIS Desktop 10.5 (Environmental Systems Research Institute, Redlands, CA, USA; https://www.esri.com) with satellite imagery basemap from Esri World Imagery (https://www.arcgis.com/home/item.html?id=10df2279f9684e4a9f6a7f08febac2a9) and Landsat 8 OLI/TIRS imagery courtesy of the U.S. Geological Survey (https://earthexplorer.usgs.gov).

**Fig. 8:**
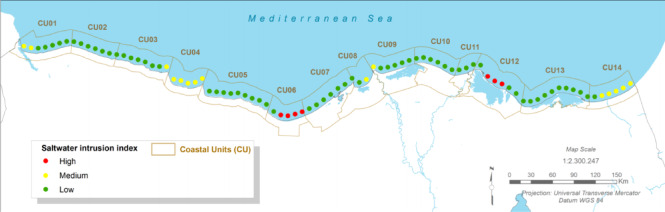
The Present Saltwater Intrusion Index (CU12: Port said). Note: Fig. 8. Present-day saltwater intrusion index for Coastal Unit 12 (Port Said). Hazard index calculated using methodologies detailed in Sect. 4.6 and Table 2. Adapted and modified from ACCNDP (2017)^12^, publicly available at: https://iczmegypt.ihcantabria.com/. Original figure created using ArcGIS Desktop 10.5 (Environmental Systems Research Institute, Redlands, CA, USA; https://www.esri.com) with satellite imagery basemap from Esri World Imagery (https://www.arcgis.com/home/item.html?id=10df2279f9684e4a9f6a7f08febac2a9) and Landsat 8 OLI/TIRS imagery courtesy of the U.S. Geological Survey (https://earthexplorer.usgs.gov).

**Fig. 9:**
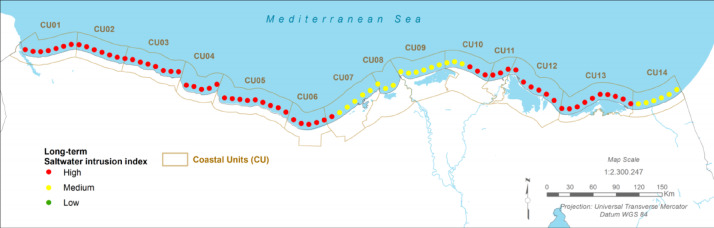
The Long-term Saltwater Intrusion Index (CU12: Port said). Note: Fig. 9. Long-term (2050–2070) saltwater intrusion index for Coastal Unit 12 (Port Said) under RCP 8.5 scenario. Hazard index calculated using methodologies detailed in Sect. 4.6 and Table 2. Adapted and modified from ACCNDP (2017)^12^, publicly available at: https://iczmegypt.ihcantabria.com/. Original figure created using ArcGIS Desktop 10.5 (Environmental Systems Research Institute, Redlands, CA, USA; https://www.esri.com) with satellite imagery basemap from Esri World Imagery (https://www.arcgis.com/home/item.html?id=10df2279f9684e4a9f6a7f08febac2a9) and Landsat 8 OLI/TIRS imagery courtesy of the U.S. Geological Survey (https://earthexplorer.usgs.gov).

**Fig. 10:**
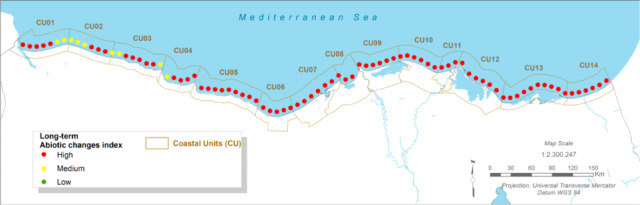
The Long Term Abiotic Stress Index (CU12: Port said). Note: Fig. 10. Long-term (2050–2070) abiotic stress index for Coastal Unit 12 (Port Said) under RCP 8.5 scenario. Hazard index calculated using methodologies detailed in Sect. 4.6 and Table 2. Adapted and modified from ACCNDP (2017)^12^, publicly available at: https://iczmegypt.ihcantabria.com/. Original figure created using ArcGIS Desktop 10.5 (Environmental Systems Research Institute, Redlands, CA, USA; https://www.esri.com) with satellite imagery basemap from Esri World Imagery (https://www.arcgis.com/home/item.html?id=10df2279f9684e4a9f6a7f08febac2a9) and Landsat 8 OLI/TIRS imagery courtesy of the U.S. Geological Survey (https://earthexplorer.usgs.gov).

**Fig. 11:**
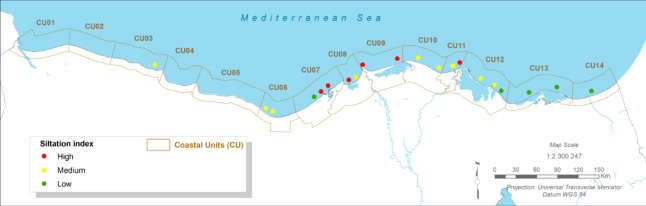
The present Siltation Index (CU12: Port said). Note: Fig. 11. Present-day siltation index for Coastal Unit 12 (Port Said). Hazard index calculated using methodologies detailed in Sect. 4.6 and Table 2. Adapted and modified from ACCNDP (2017)^12^, publicly available at: https://iczmegypt.ihcantabria.com/. Original figure created using ArcGIS Desktop 10.5 (Environmental Systems Research Institute, Redlands, CA, USA; https://www.esri.com) with satellite imagery basemap from Esri World Imagery (https://www.arcgis.com/home/item.html?id=10df2279f9684e4a9f6a7f08febac2a9) and Landsat 8 OLI/TIRS imagery courtesy of the U.S. Geological Survey (https://earthexplorer.usgs.gov).

**Fig. 12:**
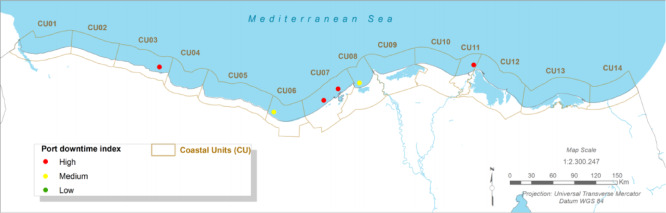
The Present Port downtime Index (CU12: Port said). Note: Fig. 12. Present-day port downtime index for Coastal Unit 12 (Port Said). Hazard index calculated using methodologies detailed in Sect. 4.6 and Table 2. Adapted and modified from ACCNDP (2017)^12^, publicly available at: https://iczmegypt.ihcantabria.com/. Original figure created using ArcGIS Desktop 10.5 (Environmental Systems Research Institute, Redlands, CA, USA; https://www.esri.com) with satellite imagery basemap from Esri World Imagery (https://www.arcgis.com/home/item.html?id=10df2279f9684e4a9f6a7f08febac2a9) and Landsat 8 OLI/TIRS imagery courtesy of the U.S. Geological Survey (https://earthexplorer.usgs.gov).

**Fig. 13:**
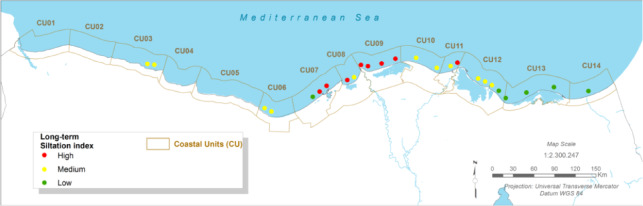
The Long-term Siltation Index (CU12: Port said). Note: Fig. 13. Long-term (2050–2070) siltation index for Coastal Unit 12 (Port Said) under RCP 8.5 scenario. Hazard index calculated using methodologies detailed in Sect. 4.6 and Table 2. Adapted and modified from ACCNDP (2017)^12^, publicly available at: https://iczmegypt.ihcantabria.com/. Original figure created using ArcGIS Desktop 10.5 (Environmental Systems Research Institute, Redlands, CA, USA; https://www.esri.com) with satellite imagery basemap from Esri World Imagery (https://www.arcgis.com/home/item.html?id=10df2279f9684e4a9f6a7f08febac2a9) and Landsat 8 OLI/TIRS imagery courtesy of the U.S. Geological Survey (https://earthexplorer.usgs.gov).

**Fig. 14:**
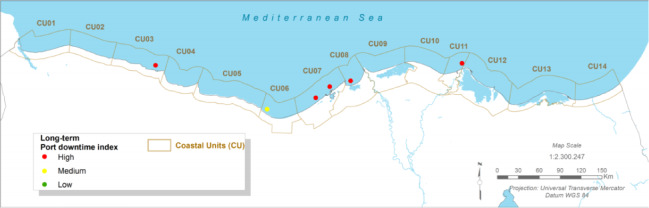
The Long-term Port downtime Index (CU12: Port said). Note: Fig. 14. Long-term (2050–2070) port downtime index for Coastal Unit 12 (Port Said) under RCP 8.5 scenario. Hazard index calculated using methodologies detailed in Sect. 4.6 and Table 2. Adapted and modified from ACCNDP (2017)^12^, publicly available at: https://iczmegypt.ihcantabria.com/. Original figure created using ArcGIS Desktop 10.5 (Environmental Systems Research Institute, Redlands, CA, USA; https://www.esri.com) with satellite imagery basemap from Esri World Imagery (https://www.arcgis.com/home/item.html?id=10df2279f9684e4a9f6a7f08febac2a9) and Landsat 8 OLI/TIRS imagery courtesy of the U.S. Geological Survey (https://earthexplorer.usgs.gov).

**Fig. 15:**
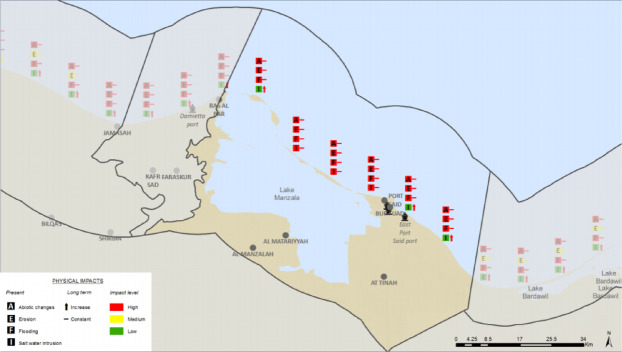
The Synthesis Physical impacts. Note: Fig. 15. Synthesis of physical impacts for Coastal Unit 12 (Port Said). Composite hazard mapping showing spatial convergence of erosion, flooding, and saltwater intrusion hazards. Adapted and modified from ACCNDP (2017)^12^, publicly available at: https://iczmegypt.ihcantabria.com/. Original figure created using ArcGIS Desktop 10.5 (Environmental Systems Research Institute, Redlands, CA, USA; https://www.esri.com) with satellite imagery basemap from Esri World Imagery (https://www.arcgis.com/home/item.html?id=10df2279f9684e4a9f6a7f08febac2a9) and Landsat 8 OLI/TIRS imagery courtesy of the U.S. Geological Survey (https://earthexplorer.usgs.gov).

**Fig. 16:**
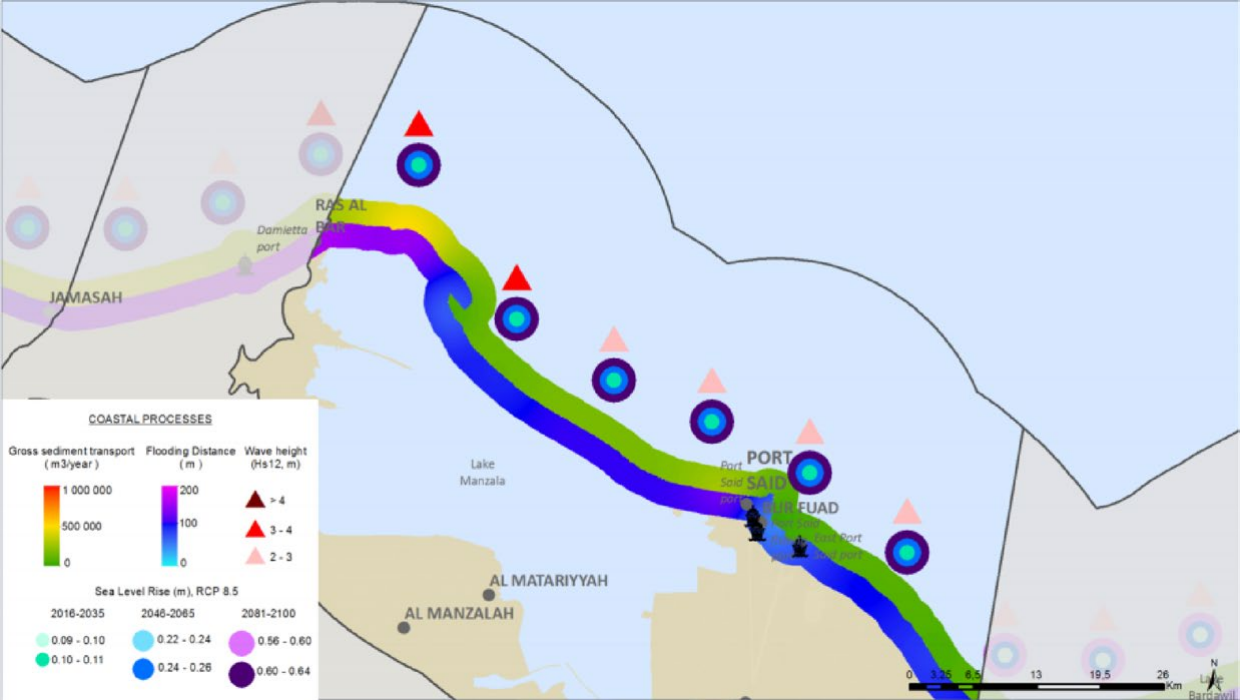
The Synthesis Coastal processes (CU12: Port said). Note: Fig. 16. Synthesis of coastal processes for Coastal Unit 12 (Port Said). Adapted and modified from ACCNDP (2017)^12^, publicly available at: https://iczmegypt.ihcantabria.com/. Original figure created using ArcGIS Desktop 10.5 (Environmental Systems Research Institute, Redlands, CA, USA; https://www.esri.com) with satellite imagery basemap from Esri World Imagery (https://www.arcgis.com/home/item.html?id=10df2279f9684e4a9f6a7f08febac2a9) and Landsat 8 OLI/TIRS imagery courtesy of the U.S. Geological Survey (https://earthexplorer.usgs.gov).

**Fig. 17:**
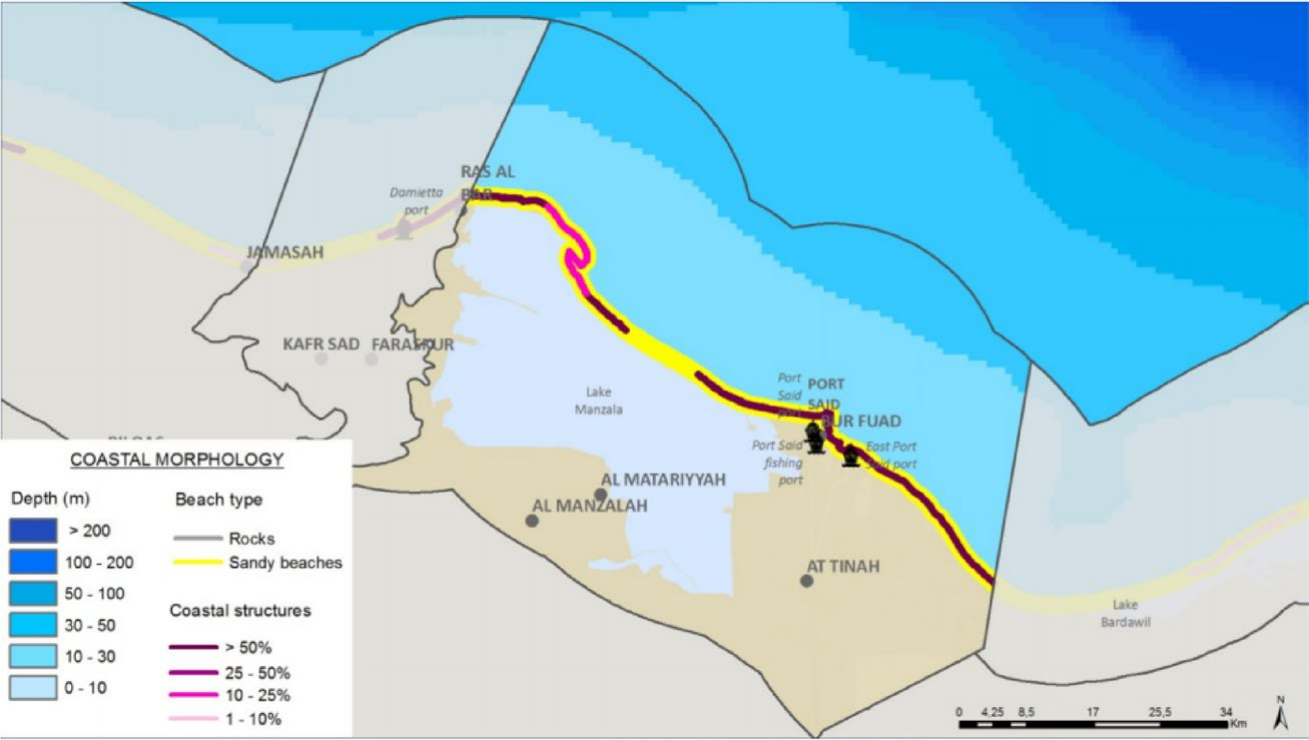
The Synthesis Coastal Morphology (CU12: Port said). Note: Fig. 17. Synthesis of coastal morphology for Coastal Unit 12 (Port Said). Adapted and modified from ACCNDP (2017)^12^, publicly available at: https://iczmegypt.ihcantabria.com/. Original figure created using ArcGIS Desktop 10.5 (Environmental Systems Research Institute, Redlands, CA, USA; https://www.esri.com) with satellite imagery basemap from Esri World Imagery (https://www.arcgis.com/home/item.html?id=10df2279f9684e4a9f6a7f08febac2a9) and Landsat 8 OLI/TIRS imagery courtesy of the U.S. Geological Survey (https://earthexplorer.usgs.gov).

**Fig. 18:**
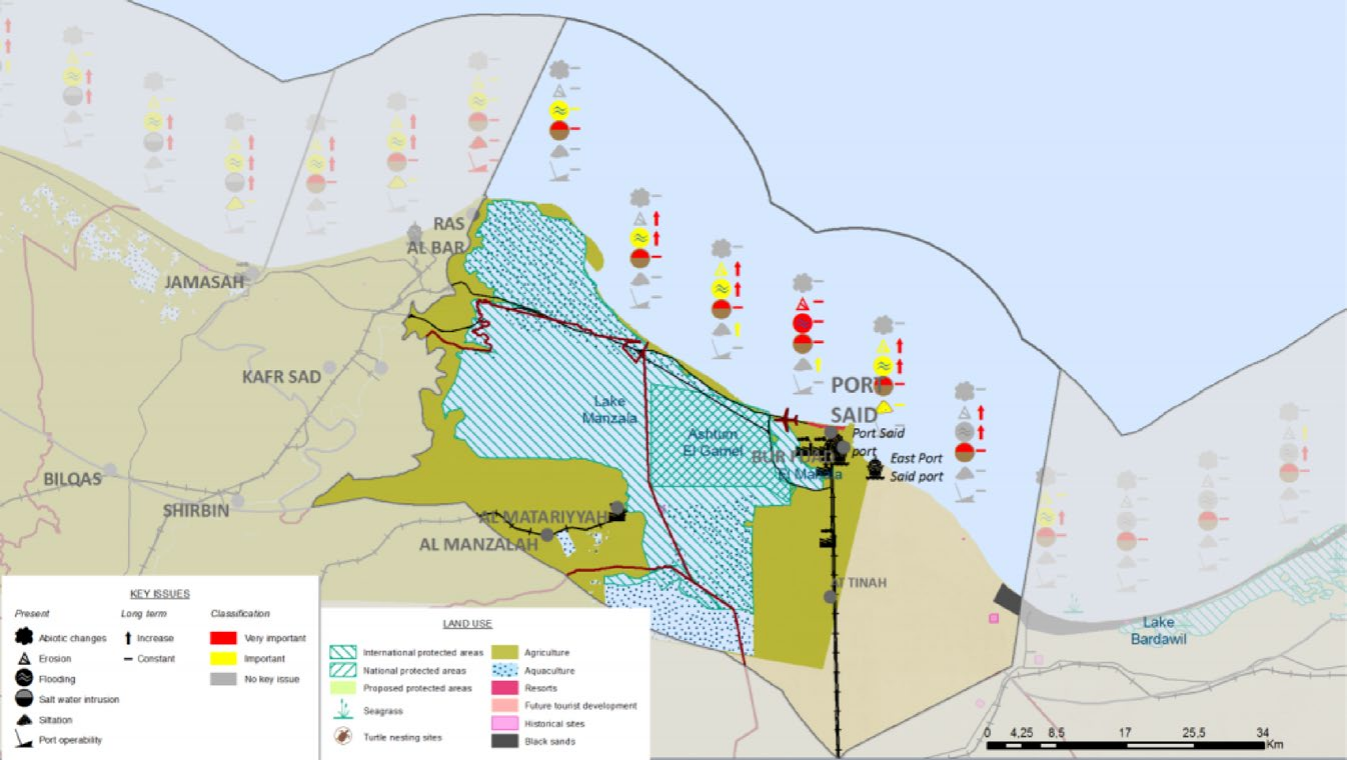
The Synthesis key Coastal issues (CU12: Port said). Note: Fig. 18. Synthesis of key coastal issues for Coastal Unit 12 (Port Said). Adapted and modified from ACCNDP (2017)^12^, publicly available at: https://iczmegypt.ihcantabria.com/. Original figure created using ArcGIS Desktop 10.5 (Environmental Systems Research Institute, Redlands, CA, USA; https://www.esri.com) with satellite imagery basemap from Esri World Imagery (https://www.arcgis.com/home/item.html?id=10df2279f9684e4a9f6a7f08febac2a9) and Landsat 8 OLI/TIRS imagery courtesy of the U.S. Geological Survey (https://earthexplorer.usgs.gov).

All spatial analyses and cartographic outputs presented in Figs. 1, 2, 3, 4, 5, 6, 7, 8, 9, 10, 11, 12, 13, 14, 15, 16, 17 and 18 were generated using ArcGIS Desktop version 10.5 (Environmental Systems Research Institute [ESRI], Redlands, California, USA; https://www.esri.com). Satellite imagery basemaps were sourced from two primary repositories: (1) Esri World Imagery (https://www.arcgis.com/home/item.html?id=10df2279f9684e4a9f6a7f08febac2a9), providing high-resolution (≤ 1 m) optical imagery for detailed coastal feature digitization and geomorphological mapping; and (2) Landsat 8 Operational Land Imager/Thermal Infrared Sensor (OLI/TIRS) imagery (30 m spatial resolution) obtained from the United States Geological Survey (USGS) EarthExplorer platform (https://earthexplorer.usgs.gov), utilized for land use/land cover classification and multi-temporal shoreline change analysis (acquisition dates: 2013–2023). Coastal boundary and administrative unit shapefiles were sourced from the Egyptian Ministry of Water Resources and Irrigation, Coastal Protection Authority, as part of the national Integrated Coastal Zone Management (ICZM) framework. The ACCNDP project outputs, including all base maps and spatial datasets, are publicly available through the Institute of Environmental Hydraulics of the University of Cantabria (IHCantabria) at: https://iczmegypt.ihcantabria.com/.

### Hydrodynamic and morphodynamic models

A suite of established empirical and analytical models was employed to quantify coastal processes, with model selection guided by data availability and demonstrated applicability to the Nile Delta context.

#### Sediment Transport:

Alongshore sediment transport rates were calculated using the Van Rijn (2002) formula, which has shown good agreement with local studies in the Nile Delta. The mass transport rate (Qt, mass, kg/s) is estimated as.

1 $${\mathrm{Qt}},{\text{mass }} = {\text{ Ksand }}\cdot{\text{ }}\left( {{\mathrm{Hs}},{\mathrm{br}}} \right)^3{\text{ }}\cdot{\text{ sin}}\left( {2\theta {\mathrm{br}}} \right)$$

where Hs, br is the breaker wave height (m), θbr is the breaking angle relative to shoreline normal (degrees), and Ksand is a calibration constant (40 kg/s/m³ for Nile Delta quartz sand)^49^. Cross-shore sediment transport was estimated using the Bailard (1982) formulation^50^.

#### Shoreline Retreat:

The long-term response of the shoreline to sea-level rise was modelled based on the principles of the Bruun rule. Horizontal retreat (R, m) is expressed as.

2 $$\mathrm{R} = \mathrm{S} \cdot \mathrm{L} / (\mathrm{h} + \mathrm{B})$$

where S is relative sea-level rise (m, incorporating both eustatic SLR and local subsidence), L is the active profile length (horizontal distance from shoreline to depth of closure, m), h is the depth of closure (h = 8 m for Port Said region based on local wave climate), and B is the berm height (B = 1.5 m for Port Said coastal barrier)^51,52^.

We acknowledge the limitations of this 2D model in the sediment-starved Nile Delta, where longshore transport is interrupted by port jetties^12,48^. To ensure scientific rigor, this study integrates the Bruun principle with 50 years of historical shoreline change data (1973–2023). By calibrating the model against observed retreat rates (5.66–12.8 m/year), the diagnosis accounts for site-specific sedimentological deficits and existing coastal protection efficacy^26,48^.

#### Coastal Flooding:

The total potential inundation level (TWL, m) was calculated as the linear superposition of astronomical tide, storm surge height derived from the GOS database, and wave setup at the shoreline.

3 $${\text{TWL }} = {\text{ }}\eta {\text{tide }} + {\text{ }}\eta {\text{surge }} + {\text{ }}\eta {\mathrm{wave}}\_{\mathrm{setup}}$$

where ηtide is astronomical tide elevation (m), ηsurge is storm surge residual (m), and ηwave_setup is wave setup (m), calculated as ηwave_setup = 0.15 · Hs, br^12^.

#### Port Downtime:

Operational downtime risk was assessed by calculating wave overtopping rates at key port structures using the formulation proposed by Owen (1980). The dimensionless mean overtopping discharge (Q*) is calculated as.

4 $${\mathrm{Q}}^*{\text{ }} = {\text{ q }}/{\text{ }}\left( {{\text{g }}\cdot{\text{ Hm}}0{\text{ }}\cdot{\text{ Tz}}} \right){\text{ }} = {\text{ a }}\cdot{\text{ exp}}\left[ { - {\text{b }}\cdot{\text{ }}\left( {{\text{Rc }}/{\text{ }}\left( {{\mathrm{Hm}}0{\text{ }}\cdot{\text{ }}\gamma {\mathrm{f}}} \right)} \right)} \right]$$

where q is mean overtopping discharge per metre width (m³/s/m), g is gravitational acceleration (9.81 m/s²), Hm0 is significant wave height at the toe (m), Tz is mean zero-crossing wave period (s), Rc is crest freeboard (m), γf is the roughness reduction factor (γf = 0.5 for rubble-mound armour), and a, b are empirical coefficients (a = 0.08, b = 20 for permeable rubble-mound structures)^53,54^.

### Spatiotemporal framework: scenarios, time slices, and coastal analysis units

To provide a structured and comparable assessment, a clear spatiotemporal framework was established.

#### Temporal Framework:

The analysis was conducted for three distinct time slices to capture the evolution of risk over time.

- **Present**: Based on a historical baseline period (1979–2015 for marine data; 1985–2015 for SST data).
- **Near-Term**: A mid-century projection centred on the period 2030–2040.
- **Long-Term**: An end-of-century projection centred on the period 2050–2070.

#### Scenarios:

To capture a range of plausible climate futures, all projections were run for two IPCC Representative Concentration Pathways.

- **RCP 4.5**: A moderate-emissions scenario assuming the implementation of climate policies that lead to a stabilisation of radiative forcing by 2100.
- **RCP 8.5**: A high-emissions, ‘business-as-usual’ scenario assuming continued growth in greenhouse gas emissions throughout the 21 st century.

#### Spatial Division:

To facilitate a spatially explicit and uniform analysis, the Port Said coastline was segmented into standardized subunits of 10 km in length. Each subunit is represented by a central ‘Coastal Point’ where all physical impact indicators were computed, allowing for a consistent, high-resolution assessment of risk variation along the coast^12^.

### Quantification of hazard indicators

To ensure methodological transparency and replicability, each of the seven selected climate drivers was quantified using a specific, measurable indicator. The methodologies are summarised in Table 2. This approach transforms abstract risks into tangible metrics that can be mapped, compared, and integrated into planning frameworks.

**Table 2:** Hazard quantification indicators and methodologies.

Hazard Driver	Indicator(s)	Methodology/Formula	Data Source(s)
Coastal Erosion	Shoreline Retreat Rate (m/yr)	Bruun rule principle calibrated with historical satellite imagery analysis (1973–2023); Long-Term Erosion indicator (LTI) combining multiple factors	Historical satellite data, bathymetry, SLR projections
Flooding	Inundation Depth (m); Flooding Distance (m)	Summation of astronomical tide, storm surge, and wave setup to determine total water level; projected onto Digital Elevation Model (DEM)	GOS database, tide gauge data, wave models, ASTER GDEM V2
Saltwater Intrusion	Groundwater Salinity (TDS in mg/L)	Hydrogeological modelling informed by aquifer characteristics and projected sea-level rise	Well data, hydrogeological maps, SLR projections
Abiotic Stress	Probability of *Cymodocea nodosa* occurrence	Species distribution model based on sea surface temperature (SST), turbidity, and salinity projections	SST projections, oceanographic data
Drought	Consecutive Dry Days (CDD)	Meteorological index counting consecutive days with precipitation < 1 mm	Climate model precipitation outputs
Heat Waves	Heat Wave Frequency & Duration	Index based on spells of days exceeding a specific temperature threshold (35 °C)	Climate model temperature outputs
Port Downtime/Siltation	Annual Hours of Inoperability; Siltation Rate (m³/yr)	Overtopping calculations (Owen 1980); longshore sediment transport models (Van Rijn 2002)	Wave models, bathymetry, port infrastructure data

### Uncertainty quantification

The diagnosis accounts for model uncertainty by presenting projections as ensemble ranges where appropriate. For sea-level rise in the Nile Delta, the 10th and 90th percentile spreads under RCP 8.5 range from 0.39 m to 1.24 m by 2100^2,55^. For temperature increases, median projections of 1.27 °C by 2030 carry an ensemble spread of 0.61 °C (10th percentile) to 2.11 °C (90th percentile)^55^. These ranges allow for risk-averse planning in the SCZONE by considering worst-case physical boundary conditions.

#### CMIP5 and CMIP6 Intercomparison:

The climate forcing utilized in this diagnosis is anchored in the CMIP5 multi-model ensemble (MME). However, recent inter-comparisons for the Egypt region indicate that CMIP6 models, which utilize Shared Socioeconomic Pathways (SSPs), generally project more severe environmental shifts^56,57^. For the economically vital northern region, CMIP6 projects a higher reduction in precipitation (10–26 mm) than CMIP5 (0–17 mm)^56^. Furthermore, CMIP6 temperature rise estimates are 0.74–1.63°C higher than CMIP5 baseline for high-emission storylines (RCP 8.5 vs. SSP 5–8.5.5)^56^. While CMIP6 models demonstrate reduced bias in seasonal variability, the ‘hot model’ issue—wherein certain models exhibit extremely high climate sensitivity—suggests that our reliance on CMIP5 provides a robust, mid-range diagnostic baseline for urban planning in Port Said^57,58^.

### Composite hazard index for critical risk triangle delineation

To identify zones of compound hazard convergence, a Composite Hazard Index (CHI) was calculated as the weighted sum of normalized hazard scores:

CHI = (E_norm · w_E) + (F_norm · w_F) + (S_norm · w_S) (5).

where E_norm, F_norm, and S_norm are normalized coastal erosion, flooding, and saltwater intrusion risk scores (0–1 scale, derived from shoreline retreat projections, TWL inundation extent, and aquifer salinity projections respectively), and w_E, w_F, w_S are weighting factors (equal weighting: w_E = w_F = w_S = 0.333). The ‘Critical Risk Triangle’ was defined as areas where CHI ≥ 0.7 (threshold based on 70th percentile of baseline hazard distribution).

## Results

This section presents the quantitative findings of the physical diagnosis, systematically detailing the present and projected impacts for each of the seven climate drivers. All future projections are presented comparatively for the near-term (2030–2040) and long-term (2050–2070) horizons under both RCP 4.5 and RCP 8.5 scenarios.

### Coastal morphodynamics: projected shoreline erosion

#### Present:

The Port Said coastline is currently characterised by a high level of erosion. Historical analysis of satellite imagery from 1973 to 2023 reveals erosion rates fluctuating between 5.66 and 12.8 m/year^48^. This erosion is particularly acute in the downdrift (eastern) sections of major coastal structures like the Port Said port entrance and the El Gamail inlet jetties, which act as barriers to the natural eastward longshore sediment transport^12^.

#### Future Projections:

The analysis projects that erosion will remain a high-impact hazard and will intensify under all future scenarios. This is driven primarily by the projected acceleration of shoreline retreat due to relative sea-level rise. For the long-term (2050–2070) under RCP 8.5, shoreline retreat is expected to be severe, leading to significant loss of land along the fragile sand barriers that protect Lake Manzala and the urban frontage. Even under the more moderate RCP 4.5 scenario, erosion issues are projected to increase, threatening existing infrastructure and narrowing the protective buffer of the remaining beaches^12^.

### Hydrodynamic hazards: coastal flooding

#### Present:

The region’s extremely low-lying topography makes it highly susceptible to coastal flooding. The baseline analysis indicates a high present-day flood risk, primarily driven by storm surges, which can elevate sea levels by up to 1.0 m along the Egyptian Mediterranean coast, in addition to astronomical tides^12,59^.

#### Future Projections:

Coastal flood risk is projected to increase dramatically across all scenarios and timeframes. Due to the combined effect of eustatic sea-level rise and high local subsidence, the flooding hazard is expected to become ‘strong’ across almost the entire coastal unit, even in the near-term (2030–2040) under the RCP 4.5 scenario^12^. Under the long-term RCP 8.5 scenario, the increase in total water level will lead to extensive and more frequent inundation of low-lying urban areas, agricultural lands, and the ecologically sensitive wetlands surrounding Lake Manzala.

### Hydrological and geochemical stress: saltwater intrusion

#### Present:

The coastal aquifer beneath Port Said is already under severe stress, with a ‘high’ degree of saltwater intrusion^12^. Hydrochemical studies in East Port Said confirm that the shallow Quaternary aquifer is highly saline, with Total Dissolved Solids (TDS) values ranging from 7,558 to 23,218 mg/L, classifying the water as Na-Cl type and indicating significant seawater intrusion^18^. This is driven by the natural hydraulic connection to the sea and exacerbated by over-extraction of groundwater for agricultural and urban use^18,60^.

#### Future Projections:

The problem of saltwater intrusion is projected to be significantly exacerbated under all future climate scenarios^12^. As relative sea level rises, the hydraulic gradient will shift, pushing the freshwater-saltwater interface further inland. This will increase the salinity of the aquifer, threatening the viability of the remaining agriculture in the region and posing a major challenge for securing a sustainable freshwater supply for the rapidly growing urban population, particularly in the new East Port Said development.

### Ecosystem vulnerability: abiotic stress on the lake manzala interface

#### Present:

The coastal and marine environment of the Port Said region is characterised by ‘high’ abiotic stress. This is a result of factors including high water salinity, pollution from land-based sources, and altered hydrodynamics. Consequently, the area is devoid of sensitive marine ecosystems like the seagrass *Posidonia oceanica*, which are important indicators of coastal health^12^.

#### Future Projections:

Future climate scenarios project a further increase in sea surface temperatures and potential changes in salinity patterns within Lake Manzala and the adjacent coastal waters^12^. These changes will intensify the existing abiotic stressors, creating conditions even more hostile to sensitive marine life. This will not only preclude any possibility of ecosystem recovery but may also lead to further degradation of the ecological functions of Lake Manzala, impacting its fisheries and biodiversity^13,61^.

### Maritime infrastructure risk: port downtime and channel siltation

#### Present:

The Port of Port Said and the northern entrance to the Suez Canal are critical economic assets. Currently, operations face ‘medium’ siltation challenges in key channels like the El Gamail strait and the port entrance, requiring regular maintenance dredging^12^. More significantly, port operations are susceptible to downtime caused by wave overtopping of breakwaters and quays during severe storm events^12,62^.

#### Future Projections:

The analysis indicates that future changes in wave climate are unlikely to cause a significant increase in longshore sediment transport rates (< 1–2%), meaning that baseline siltation issues are not expected to worsen dramatically^12^. However, the risk of operational downtime due to weather is projected to increase. As sea levels rise, the effective height of coastal defence structures is reduced, allowing larger waves to overtop them more frequently. This will lead to an increase in the number of hours per year that port operations must be suspended for safety reasons, directly impacting the efficiency and reliability of this vital global trade artery^12,62^.

### Atmospheric hazards: drought and heat waves

#### Drought:

Analysis of the Consecutive Dry Days (CDD) index indicates a moderate increase in drought frequency and duration across both RCP scenarios. Under RCP 8.5, the long-term projection shows an increase in CDD of approximately 15–20 days per year relative to the historical baseline, with implications for agricultural water demand and urban water supply security.

#### Heat Waves:

Heat wave frequency and duration are projected to increase substantially, particularly under RCP 8.5. The number of days exceeding the 35 °C threshold is expected to increase by 30–40 days annually by 2050–2070, posing significant risks to public health and energy infrastructure. These projections are consistent with recent analyses of heat wave trends along the Egyptian Mediterranean coast^63^.

### Synthesis: mapping compound hazard zones

Synthesizing the spatial results for the most critical hazards reveals a clear pattern of compounding risk. When the projected zones of high erosion, high flooding, and high saltwater intrusion are overlaid, a ‘Critical Risk Triangle’ emerges. This hotspot of multiple, interacting hazards is most pronounced in the eastern part of the study area, encompassing the coastal strip from Port Said city eastward across the Sahl El Tina plain.

This geographical convergence of severe physical risks is deeply concerning because it directly overlaps with the strategic location designated for the new East Port Said city and its associated industrial and logistical zones. The decision to site a massive urban expansion, intended to house over a million people and critical economic infrastructure, in the area of highest identified compound risk points to a profound misalignment between current development planning and future climate realities. These hazards are not independent; they are mutually reinforcing. Coastal erosion of the protective sand barrier directly increases exposure to flooding. Major flood events, in turn, can accelerate saltwater intrusion by over-washing the low-lying land and directly recharging the shallow aquifer with saline water. Placing a major new urban centre in this high-risk zone without unprecedented levels of engineered protection and adaptive management risks creating a large-scale, long-term case of maladaptation, locking in extreme vulnerability and escalating the future economic and social costs of climate change.

## Discussion

### The amplifying effect of compound hazards on urban vulnerability

The results of this physical diagnosis underscore a critical reality for coastal cities like Port Said: climate risk is not a simple summation of individual hazards but a product of their complex and dynamic interactions. The concept of compound events—where multiple climate drivers occur simultaneously or in close succession—is central to understanding the true scale of urban vulnerability^3,4^. For Port Said, a scenario involving a severe storm surge (hydrodynamic hazard) coinciding with an extreme precipitation event (atmospheric hazard) could overwhelm both coastal defence systems and urban drainage networks simultaneously, leading to a flooding catastrophe far greater than either event would cause in isolation. Similarly, the slow-onset pressures of sea-level rise and land subsidence create a baseline of heightened vulnerability that amplifies the impact of every subsequent storm event^58,64^. This non-linear amplification means that conventional, single-hazard engineering and planning approaches are likely to be insufficient, necessitating a paradigm shift towards integrated, multi-risk management frameworks.

### Port Said in a Global Context: Comparative Insights from International Delta Cities

Situating Port Said’s challenges within a global context provides valuable perspective on potential adaptation pathways.

#### Rotterdam, The Netherlands:

Often cited as a global leader in delta management, Rotterdam offers a model of proactive and integrated adaptation. Its ‘Climate Proof’ strategy combines robust hard infrastructure (e.g., the Maeslantkering storm surge barrier) with innovative, nature-based, and adaptive urban design solutions like ‘water squares’ for temporary storm water storage, extensive green roofs, and the ‘Room for the River’ programme^65–67^. Rotterdam’s success is rooted in a long-term vision, strong institutional coordination between water boards and the municipality, and sustained public investment, providing a benchmark for what is possible with strategic foresight and governance.

#### Ho Chi Minh City, Vietnam:

As a rapidly urbanising delta city in the Global South, Ho Chi Minh City shares many of Port Said’s challenges, including extreme vulnerability to flooding, land subsidence, and the pressures of rapid development^68,69^. Its experience highlights the difficulties of implementing adaptation measures in a context of limited financial resources, fragmented governance, and competing development priorities, reflecting the common struggles of many cities in similar developmental and geographical situations.

#### Lagos, Nigeria:

Representing a large, rapidly growing coastal city in Africa, Lagos faces extreme exposure to sea-level rise and coastal flooding^58^. Its case illustrates the profound social equity dimensions of climate vulnerability, where informal settlements often bear the brunt of climate impacts. This comparison underscores the importance of ensuring that adaptation strategies in Port Said are inclusive and address the vulnerabilities of all socioeconomic groups.

### Systemic implications for critical infrastructure, water security, and economic Stability

The physical risks diagnosed in this study have far-reaching systemic implications. The operational integrity of the Suez Canal and its associated ports is paramount not only for the Egyptian economy but for global maritime trade. Increased downtime due to weather, coupled with the long-term threat of inundation to port infrastructure, represents a significant economic risk^62,70,71^. Furthermore, the viability of the massive investment in the East Port Said development is called into question by its location within a compound hazard hotspot. Without a fundamental re-evaluation of its design and resilience strategy, it risks becoming a stranded asset. The escalating threat of saltwater intrusion poses an existential challenge to water security, undermining the potential for local agriculture and increasing the cost and energy intensity of providing potable water to a growing urban population. Collectively, these impacts threaten the long-term economic stability and sustainable development of the entire region.

#### Socioeconomic Exposure and Asset Risk:

The identification of the ‘Critical Risk Triangle’ demonstrates a direct threat to the regional expansion strategy. Spatial overlay of hazard zones against the East Port Said Master Plan reveals that approximately USD 35 billion in property assets and tourism potential is situated in high-vulnerability flood paths^72^. Furthermore, the Suez Canal Economic Zone has attracted USD 15 billion in investments (70% foreign), much of which is geographically concentrated in the diagnosed ‘strong hazard’ areas^10,14^. Without adaptive structural heightening, the number of hours of port downtime could increase by 15–20%, impacting a trade artery that handles 12% of global commerce^9^.

### Limitations, uncertainties, and avenues for future research

This study, while comprehensive, is subject to certain limitations and uncertainties inherent in climate change impact assessment. Projections are based on GCMs, which have inherent biases and uncertainties, particularly regarding regional precipitation patterns. The use of empirical models like the Bruun rule for shoreline retreat involves simplifying assumptions about coastal processes, although we have mitigated this limitation through calibration against 50 years of observed shoreline change data. The analysis would benefit from higher-resolution topographic and bathymetric data to refine inundation modelling; future research should prioritize acquisition of LiDAR-derived elevation data for high-resolution flood path modeling.

While this study quantifies exposed assets, it does not perform a full cost-benefit analysis of adaptation versus retreat; developing a robust business case for resilience investment in Port Said remains a critical next step for sustainability science. Additionally, future socio-ecological modeling should explore the impacts of abiotic stress on artisanal fisheries, ensuring that adaptation pathways remain inclusive and equitable.

Future research should build upon this physical diagnosis in several key areas. There is a critical need for integrated socio-ecological modelling to assess the cascading impacts on human populations and ecosystems. Detailed economic vulnerability assessments are required to quantify the potential financial losses under different scenarios and to build a business case for proactive adaptation investment. Finally, in-depth governance analysis is needed to identify the institutional barriers and enablers for implementing the recommended adaptation strategies.

### Theoretical implications for urban sustainability transitions

The case of Port Said provides a compelling empirical illustration of the core concepts of urban sustainability transitions theory^29,73^. This body of theory posits that addressing persistent, complex societal challenges like climate change requires fundamental, non-linear transformations in dominant socio-technical systems, including urban form, infrastructure, and governance regimes^30,74^. The current development trajectory of Port Said, particularly the expansion into high-risk coastal zones, can be conceptualised as reinforcing an existing, vulnerable paradigm—a potential ‘lock-in’ to a future of escalating risk and reactive crisis management. The findings of this physical diagnosis highlight the urgent need for a deliberate ‘transition pathway’ towards a new regime of climate-resilient and sustainable urbanism. This would involve not just technological fixes but a fundamental shift in planning philosophies, institutional arrangements, and investment priorities.

### Robustness of projections and contribution to knowledge

The methodological approach adopted in this study contributes to the growing body of knowledge on climate risk assessment in data-scarce environments. By integrating publicly available global reanalysis datasets (GOW, GOS, GOT) with downscaled CMIP5 projections and local validation against tide gauge records and historical shoreline observations, we demonstrate a transferable framework that can be applied in other medium-sized coastal cities with limited monitoring infrastructure. The explicit treatment of land subsidence as a risk multiplier—quantified at 4.1–5.3 mm/year for Port Said—provides a critical refinement over assessments that consider only eustatic sea-level rise, effectively reducing the perceived adaptation window and heightening the urgency for action.

The validation metrics reported (Pearson *r* = 0.83, RMSE = 0.11 m for surge reanalysis) confirm that global datasets can provide reliable baselines for local-scale diagnosis when appropriately calibrated. Furthermore, the comparative analysis of CMIP5 and CMIP6 projections suggests that our reliance on CMIP5 provides a conservative, mid-range estimate that may underestimate future warming and precipitation deficits under more recent model iterations, lending additional weight to the precautionary principle in adaptation planning.

## Policy implications for urban sustainability in port said

Translating the scientific findings of this physical diagnosis into actionable policy is essential for guiding Port Said towards a sustainable and resilient future. The following recommendations are designed to be integrated into existing urban planning and governance structures, aligning with national strategic objectives. Table 3 provides a summary framework linking identified vulnerabilities to specific adaptation strategies and relevant national policies.

**Table 3:** Framework for action: linking adaptation strategies to key vulnerabilities.

Identified Vulnerability	Affected Sectors	Recommended Adaptation Strategy	Alignment with National Policy
High Coastal Erosion & Flooding	Urban Development, Infrastructure, Tourism	Risk-Informed Zoning: Implement stringent coastal setback policies and restrict new construction in high-risk zones. Nature-Based Solutions: Initiate large-scale dune restoration and wetland rehabilitation projects along the Lake Manzala barrier to act as natural buffers.	Egypt Vision 2030 (Environment Pillar), National Adaptation Plan
High Saltwater Intrusion	Water Supply, Agriculture	Integrated Water Management: Develop a strategy for managed aquifer recharge and invest in non-conventional water sources (desalination, wastewater recycling) for new urban areas.	National Water Resources Plan, Egypt Vision 2030
Increased Port Downtime	Maritime Trade, Logistics, Suez Canal Operations	Infrastructure Resilience: Upgrade and heighten breakwaters and seawalls using adaptive design principles. Develop advanced early warning systems for storm events to optimize port operations and minimize downtime.	Suez Canal Economic Zone (SCZONE) Sustainability Strategy
Intensified Heat Waves	Public Health, Energy	Urban Greening & Cool Infrastructure: Mandate the use of green roofs, cool pavements, and increase urban green spaces in all new developments, particularly in East Port Said.	National Climate Change Strategy, Egypt Vision 2030

### Towards risk-informed spatial planning and adaptive governance

The most critical policy implication is the need to embed the findings of this and future climate risk assessments directly into statutory land-use planning and zoning regulations. The compound hazard maps produced in this study should serve as a foundational layer for all future development decisions, particularly concerning the ongoing expansion of East Port Said. An adaptive governance approach is required, wherein development plans are not static but are subject to regular review and revision in light of new climate science and monitoring data. This includes establishing clear, legally enforceable coastal construction setback lines to create a buffer against future erosion and flooding.

### Championing nature-based and hybrid solutions for coastal defence

While hard engineering structures will remain a necessary component of Port Said’s coastal defence, a strategic shift towards nature-based solutions (NbS) and hybrid approaches is strongly recommended. The restoration and protection of the sand dune systems and the wetlands along the Lake Manzala barrier can provide a highly effective and adaptive first line of defence against storm surges and coastal flooding. These green infrastructure approaches offer significant co-benefits, including enhancing biodiversity, improving water quality, and creating recreational opportunities, thus contributing to multiple sustainability goals simultaneously.

### Enhancing infrastructure resilience and operational preparedness

For critical infrastructure that cannot be relocated, resilience must be enhanced through structural upgrades and improved operational protocols. Port infrastructure, including breakwaters and quays, should be assessed and retrofitted based on future sea-level and storm intensity projections, incorporating principles of adaptive design that allow for future heightening. The development and implementation of sophisticated multi-hazard early warning systems are crucial. These systems, providing timely forecasts for both storm surges and extreme heat waves, would enable port authorities to take preemptive action to secure assets and adjust operations, and allow municipal authorities to warn and protect vulnerable populations.

### Aligning adaptation pathways with national strategic visions

To ensure political traction and facilitate implementation, all adaptation efforts in Port Said must be explicitly aligned with Egypt’s overarching national development strategies. Recommendations should be framed as essential actions for achieving the goals of Egypt Vision 2030, particularly its pillar on environmental sustainability and its commitment to enhancing resilience to natural hazards^75,76^. Interventions should also be presented as integral to the successful implementation of Egypt’s National Adaptation Plan (NAP) and National Climate Change Strategy^77^. For port-related measures, alignment with the Suez Canal Economic Zone (SCZONE) sustainability strategy, which includes a vision for a ‘Green Canal’, will be critical for securing investment and institutional support^78,79^. By demonstrating how climate resilience underpins economic competitiveness and social well-being, adaptation can be mainstreamed from a niche environmental concern into a core component of national development policy.

## Conclusions

This study has provided a comprehensive physical diagnosis of the compound climate risks facing the Port Said Urban Region, revealing a landscape of severe and accelerating vulnerability. Our principal finding is the identification of a critical misalignment between the region’s highest-risk zones—characterised by a convergence of extreme erosion, flooding, and saltwater intrusion hazards—and its most ambitious urban and industrial development plans. The region’s exceptionally high rate of land subsidence acts as a potent risk multiplier, drastically shortening the timeline for adaptation. The scientific significance of this work lies in its integrated, quantitative approach, which moves beyond single-hazard analysis to reveal the systemic nature of climate risk in a complex deltaic setting.

The ‘physical diagnosis’ framework developed and applied in this paper represents a methodologically robust and replicable approach for assessing climate risk in data-scarce environments. By combining publicly available global reanalysis data and climate projections with established analytical models—and crucially, validating these against local observational records—the methodology provides a transparent and transferable tool for other medium-sized coastal cities, particularly in the Mediterranean and the wider Global South. It offers a pathway for these cities to develop a foundational, evidence-based understanding of their climate vulnerabilities, even in the absence of extensive local monitoring networks.

Port Said stands at a critical crossroads. The current trajectory, characterised by rapid development in zones of extreme climate risk, places the city on a path toward potential large-scale maladaptation, with the prospect of locking in immense future social and economic costs. However, this diagnosis also illuminates an alternative path. By leveraging this scientific understanding to fundamentally reorient its spatial planning, invest in a portfolio of grey and green infrastructure, and embed adaptive management into its governance structures, Port Said has the opportunity to pivot. It can transform from a case study of impending vulnerability into a regional and global paradigm for proactive, science-informed, and sustainable coastal resilience, demonstrating how a globally strategic city can secure its future in the face of a changing climate.

While providing an exhaustive physical baseline, this diagnosis is subject to several limitations. First, the use of 30 m resolution DEMs may under-represent complex urban drainage paths; future research should prioritize LiDAR data for high-resolution flood path modeling. Second, the Bruun Rule’s 2D assumptions have been mitigated via historical calibration, but 3D morphodynamic simulations are required to evaluate the long-term efficacy of specific SCZONE coastal defense designs. Third, while this study quantifies exposed assets, it does not perform a full cost-benefit analysis of adaptation versus retreat; developing a robust business case for resilience investment in Port Said remains a critical next step for sustainability science. Finally, future socio-ecological modeling should explore the impacts of abiotic stress on artisanal fisheries, ensuring that adaptation pathways remain inclusive and equitable.

## Data Availability

The datasets used and/or analyzed during the current study are available from the corresponding author on reasonable request.
